# Resveratrol-Induced Changes in MicroRNA Expression in Primary Human Fibroblasts Harboring Carnitine-Palmitoyl Transferase-2 Gene Mutation, Leading to Fatty Acid Oxidation Deficiency

**DOI:** 10.3390/molecules23010007

**Published:** 2017-12-22

**Authors:** Virginie Aires, Dominique Delmas, Fatima Djouadi, Jean Bastin, Mustapha Cherkaoui-Malki, Norbert Latruffe

**Affiliations:** 1 Laboratory BioPeroxIL, University of Bourgogne-Franche Comté, 6 Blvd Gabriel, 21000 Dijon, France; virginie.aires02@u-bourgogne.fr (V.A.); ddelmas@u-bourgogne.fr (D.D.); malki@u-bourgogne.fr (M.C.-M.); 2 INSERM UMR 866, Blvd Jeanne d’Arc, 21490 Dijon, France; 3 INSERM U1124, Université Paris-Descartes, Rue des Saints-Pères, 75000 Paris, France; Fatima.djouadi@inserm.fr (F.D.); jean.bastin@inserm.fr (J.B.)

**Keywords:** resveratrol, miRNA level, *CPT2*-deficient cells

## Abstract

Carnitine palmitoyltransferase-2 (*CPT2*) is a mitochondrial enzyme involved in long-chain fatty acid entry into mitochondria for their β-oxidation and energy production. Two phenotypes are associated with the extremely reduced *CPT2* activity in genetically deficient patients: neonatal lethality or, in milder forms, myopathy. Resveratrol (RSV) is a phytophenol produced by grape plant in response to biotic or abiotic stresses that displays anti-oxidant properties, in particular through AP-1, NFκB, STAT-3, and COX pathways. Some beneficiary effects of RSV are due to its modulation of microRNA (miRNA) expression. RSV can enhance residual *CPT2* activities in human fibroblasts derived from *CPT2*-deficient patients and restores normal fatty acid oxidation rates likely through stimulation of mitochondrial biogenesis. Here, we report changes in miRNA expression linked to *CPT2*-deficiency, and we identify miRNAs whose expression changed following RSV treatment of control or *CPT2*-deficient fibroblasts isolated from patients. Our findings suggest that RSV consumption might exert beneficiary effects in patients with *CPT2*-deficiency.

## 1. Introduction

Resveratrol (RSV, trans-3,5,4′-trihydroxystilbene) is a phytoalexin produced by numerous plants in response to abiotic or biotic stress [1,2,3]. This polyphenol compound admittedly protects humans against various diseases (cardiovascular and inflammation-associated pathologies, infection, cancer, neurodegenerescence, aging, etc.) through the modulation of several signaling pathways, including those mediated by transcription factors AP-1 [4], NFκB, and STAT-3 [5] or the COX enzyme [6]. RSV has been shown to improve residual β-oxidation in primary human fibroblasts from *CTP2*-deficient patients by restoring normal fatty acid oxidation rates [7].

MicroRNAs are short, non-coding regulatory microRNAs present in plants, animals, and viruses. To date, more than 1500 miRNAs have been identified in humans. MiRNA primarily controls mRNA translation and stability. Due to their ability to regulate several hundred transcripts directly or indirectly through targeting components of key regulatory pathways, miRNAs behave as master regulators that impact all aspects of cell homeostasis and functions. Many miRNAs are considered either as tumor suppressor or onco-miRs, depending on the cellular context [8].

There is now a substantial amount of literature on miRNAs, including a few studies that have addressed the differential effects of polyphenols on miRNA expression [8,9,10,11,12,13]. In 2010, we demonstrated the regulatory effect of RSV on the expression of miRNAs involved in macrophage-associated inflammatory response [8] as well as on the expression of components of the TGFβ regulatory pathway in colon cancer cell lines [14]. Interestingly, in 2013, Milenkovic et al. [10] established that the expression of more than 100 miRNAs is modulated by polyphenols. These authors reported that various different polyphenols have both common and specific miRNA targets. Indeed, in mouse livers, over 137 miRNAs are modulated by phytophenols of the stilbenoids family (including resveratrol). While stilbenoids modulated the expression of 87 of these 137 miRNAs, 24 other miRNAs were targets of flavonoids, 6 of phenolic acids, and 20 of curcuminoids. This analysis further confirms that the different classes of polyphenols not only share common properties (as anti-oxidant for instance) but also have their own specific effects due to their unique chemical structure and reactivity and their differential effects on gene expression, especially miRNAs.

Presently, more than a hundred papers have confirmed that the effect of RSV in the prevention or treatment of various diseases, including prostate cancer [15], melanoma [16], breast cancer [17], lung tumors [18], white adipogenesis [19], liver steatosis [20], inflammation [21,22], neurodegenerative disorders [23], and osteoporosis [24], were mediated by miRNAs.

In the present study, we report the RSV-induced modulation of energy metabolism miRNA in human cells harboring mitochondrial fatty acid ß-oxidation-deficiency as a result of carnitine-palmitoyl transferase 2 (*CPT2*) gene mutations. *CPT2* is a mitochondrial inner membrane enzyme playing an essential role in the transfer of fatty acids from the cytosol to the mitochondrial matrix. In 2011, Bastin et al. [7] showed that RSV is able to enhance residual *CPT2* activity in human fibroblasts derived from patients harboring the muscular form of CTP2 deficiency and can restore near-normal fatty acids oxidation rates, opening potential clinical perspectives to successfully treat the *CPT2*-deficiency-associated myopathy. This stimulation was also observed with other analogues of the stilbene family [25]. In this study, we compared miRNA expression in control and *CPT2*-deficient primary human fibroblasts and analyzed RSV’s impact on miRNA expression in both cell lines.

## 2. Results and Discussion

### 2.1. Changes in miRNA Expression Associated with Mitochondrial CPT2-Deficiency in Primary Human Fibroblasts

Table 1 shows that *CPT2*-deficiency was associated with significant changes in the levels of 51 miRNA in patient fibroblasts. More specifically, *CPT2*-deficient fibroblasts showed 13 upregulated miRNAs (with an 11-fold increase for *miR-301* in particular) and 38 downregulated miRNAs, including 3 miRNAs from the *miR-let-7* family. The multiplicity of miRNA target transcripts suggests that the fibroblast transcriptome might be widely affected by *CPT2*-deficiency.

### 2.2. RSV-Induced Changes in miRNA Expression in Control and CPT2-Deficient Primary Human Fibroblasts

Table 2 shows changes in miRNA expression induced by RSV treatment in both control and *CPT2*-deficient fibroblasts. Twelve miRNAs were upregulated and 24 miRNAs downregulated in control fibroblasts, versus 8 miRNAs upregulated and 16 miRNAs downregulated in *CPT2*-deficient fibroblasts. Of note, *miR-566* and *miR-23a,b* were downregulated in both control and patient fibroblasts following RSV treatment, suggesting that these changes might result from RSV specific action on factors controlling transcription and/or maturation of these two miRNAs, irrespective of cell genotype or of energy metabolism deficiency. On the other hand, *miR-550-1,2* was upregulated and *miR-let7-a3* was downregulated in both untreated *CPT2*-deficient fibroblasts (Table 1) and RSV-treated control fibroblasts (Table 2). If one considers that RSV exerts beneficial effects on the cell, this suggests that the changes in expression of these two miRNAs might be advantageous in both cases. Finally, the expression of other miRNAs changed in both untreated and RSV-treated *CPT2*-deficient fibroblast. Thus, *miR-181a2,d*, *miR-let7d*, and *miR-146a* were downregulated in both untreated and RSV-treated *CPT2*-deficient fibroblast (Table 1 and Table 2). This suggests that the upregulation of these three miRNAs might provide *CPT2*-deficient fibroblasts with an increased ability to survive with reduced catabolism of long chain fatty acids.

### 2.3. Mirna Whose Expression Changed in CPT2-Deficient Primary Fibroblasts, Regardless of RSV Treatment, Target Pathways Involved in Fatty Acid Oxidation

We have shown in previous studies that treatment by resveratrol similar to that used in the present study induced a dose-dependent increase in fatty acid oxidation in *CPT2*-deficient patients [25]. Furthermore, this dose of resveratrol was shown to correct not only *CPT2* deficiency, but also other fatty acid oxidation and respiratory chain deficiencies [7,26,27]. In all these experiments, the human fibroblasts did not exhibit growth changes or increased mortality. It was thus essential to use the same treatment conditions in order to investigate the changes in microRNA expression in response to resveratrol. We also showed previously that treating mouse RAW264.7 macrophages with a 10 to 100 μM range of resveratrol concentrations decreased JunB expression as well as AP-1 activity in a dose-dependent manner (Supplementary figure 3 in the manuscript of Tili et al. [4]). Thus, we believe that treatment by 75 μM resveratrol, despite representing a high concentration, should not have caused any bias in our microarrays analyses.

The marked beneficial effects of treatment on fatty acid oxidation in the patient fibroblasts could suggest that resveratrol directly regulates *CPT2* expression. However, the precise signaling pathway(s) by which RSV targets fatty acid oxidation in the context of *CPT2*-deficiency is still a matter of debate. Several polyphenols, including RSV have been shown to increase the activity and gene expression of SIRT1 accompanied by the increase in CPT1 mRNA encoding the rate-limiting enzyme of mitochondrial fatty acid oxidation [28]. SIRT1-dependent de-acetylation of PGC-1α leads to the transcriptional co-activation of nuclear and mitochondrial genes encoding for proteins promoting mitochondrial biogenesis, oxidative phosphorylation and energy production. On the other hand, SIRT3 mediates direct activation of proteins implicated in oxidative phosphorylation, tricarboxylic acid (TCA) cycle and fatty-acid oxidation, in addition to an indirect activation of PGC-1α and AMP-activated protein kinase (AMPK). SIRT1 is required for the activation of AMPK, which enhances energy-production through glucose transport, fatty acid oxidation, or mitochondrial biogenesis [29,30]. The action of resveratrol to correct *CPT2*-deficiency might therefore involve SIRT1, however, definite evidence based on silencing SIRT1 expression in patient fibroblasts is lacking. Altogether, there is a general consensus in the literature supporting RSV effects being mediated through an AMPK/SIRT1/PGC-1α pathway [31]. It has also been suggested that RSV effects might also occur through the estrogen receptor (ER), which RSV can bind and activate [23]. We therefore examined whether the 3′-untranslated regions of genes implicated in the two above pathways contain consensus target sites for those miRNAs whose expression changed either in untreated *CPT2*-deficient fibroblasts as compared with control fibroblasts (Table 1) or in *CPT*2-deficient fibroblasts treated with RSV as compared with DMSO-treated *CPT2*-deficient fibroblasts (Table 2).

The general mechanism associated with microRNA action involves the reduced expression of their target genes. Using the Targetscan software (www.targetscan.com), we found consensus target sequences for miRNAs that were upregulated, as well as for miRNAs that were downregulated, in untreated *CPT2*-deficient fibroblasts (Table 3). In particular, *miR-483*, the miRNA that increased with the lowest *P* value in Table 1, targeted seven genes of the above pathways. It was followed by *miR-449b*, *miR-371* (with often multiple target sequences in the same transcripts), and *miR-9* (6 genes targeted) and then by *miR-539* and *miR-301* (5 and 4 genes targeted, respectively). In contrast, except for *miR-181a2,c* (seven genes targeted), the miRNAs downregulated in untreated *CPT2*-deficient fibroblasts did not target more than 4 genes (*miR-211* and *miR-126a-5*) (Table 3), and, except *miR-211*, were not among the miRNAs that changed with the lowest *P* value in Table 1. Although it would be almost impossible to measure the relative effects of these miRNAs on the expression of each of these putative target genes, the above observation suggests that the effects of upregulated miRNAs as a whole might be greater than those of downregulated miRNAs, leading to reduced levels of expression of their respective target gene and, as a consequence, reduced fatty acid oxidation.

Consensus target sites in the 3’-untranslated regions of the same genes for miRNAs that were upregulated or downregulated following RSV treatment of *CPT2*-deficient fibroblasts (Table 2) show a similar distribution. While seven of the above genes are potential targets of *miR-199a1-5p* and 5 are potential targets of *miR-337* (both upregulated following RSV treatment). Table 3 also shows that seven genes of the above pathways are also putative targets of *miR-181d*, and five of them are also targets of *miR-20b*, *miR-17-5p*, *miR-26a*, and *miR-23a* (all of these miRNAs being downregulated following RSV treatment). Given that the 3′-untranslated regions of several of these genes contains more than one consensus target site for *miR-20b*, *miR-17-5p*, or other miRNAs, it is likely that RSV may change the expression of genes that encode factors implicated in these two pathways, and therefore fatty acid oxidation, through both miRNA-dependent and miRNA-independent mechanisms. Finally, as miRNAs that changed following RSV treatment of control fibroblasts are fairly different from those that changed following RSV treatment of *CPT2*-deficient fibroblasts (Table 2), it is possible that RSV effects on miRNA expression might depend on other factors—factors most likely implicated in modulating the activity of fatty acid oxidation in mitochondria.

In conclusion, this paper is the first to report changes in microRNA expression associated with *CPT2*-deficiency in human fibroblasts and sheds some new light on potential beneficial effects of RSV through modifying miRNA expression. In particular, it appears likely that changes in miRNA levels in *CPT2*-deficient cells might, at least in part, be involved in abnormal fatty acid oxidation. The emerging role of microRNAs in lipid metabolism has been emphasized in recent reviews [32] reporting that miRNAs are critical regulators of lipid synthesis, fatty acid ß-oxidation, and lipoprotein metabolism. Changes in the expression of crucial miRNAs can impact gene regulatory network, driving to metabolic syndrome and its related pathologies. This review introduced epigenetic and transcriptional regulation of miRNA expression, especially *miR-378* (controlling *FABP7*, *IGFBP3*, *PDCD4*, and *PPAR-α* mRNA expression) and *miR-21* (controlling *CRAT*, *MED13*, *ERRγ*, *GABP1*, and *IGF1α* mRNA expression). In this paper, we found that *miR-378* was downregulated by RSV in control fibroblasts and that *miR-21* was downregulated by RSV in *CPT2*-deficient fibroblasts. In addition, *miR-21* could putatively target *NRF1* mRNAs, which encode a transcription factor implicated in respiratory control (Table 3).

Further studies will be required to assess the impact of these changes in miRNA expression on RSV-induced stimulation of mitochondrial fatty acid oxidation in *CPT2*-deficient cells and to identify factors that mediate these RSV effects.

## 3. Materials and Methods

### 3.1. Primary Human Fibroblasts and Cell Treatments

*CPT2*-deficient and control human skin fibroblasts used in this study have been described previously [5]. Point mutations and genotypes of the cells are the following: nucleotides changes, c.338C > T and c.371G > A, and consequently amino acid changes, S113L and R124Q. For cell treatment, a medium of Ham’s F10 media containing glutamine, 12% fetal bovine serum, 100 U/mL penicillin, and 0.1 mg/mL streptomycin was removed and replaced with fresh medium containing either the vehicle, DMSO 0.1%, or 75 μM resveratrol (RSV). Cells were subsequently cultivated for 72 h before RNA extraction.

### 3.2. RNA Extraction, Purification, and Micro RNAs Screening and Analysis

RNAs extracted with TRIzol (Invitrogen) were subsequently subjected to DNase digestion (Turbo-DNase-Ambion), as previously described [2]. MiRNA microarrays were analyzed as previously described [2]. Four independent repeats (i.e., cell cultures) were used for each group.

### 3.3. RNA Labeling and Micro-Arrays

Five micrograms of total RNA were labeled by reverse transcription at 37 °C for 90 min using a biotin-labeled rand-octomer oligo primer. An RT reaction mix was further denatured in 0.5 N NaOH/1 mM EDTA at 65 °C for 15 min and neutralized by 1 M Tris HCl pH 7.6. Biotin signal was detected with an Alexa 647-Streptavidin conjugate. Chips were hybridized on Tecan HS 4800 hybridization station. Chips were pre-hybridized at 25 °C for 30 min in the buffer: 6× SSPE/30% formamide/1× Denhardt’s solution. Chips were further hybridized with a labeled target in 6× SSPE/30% formamide at 25 °C for 18 h. Hybridization and post-hybridization washing was conducted in 0.75× TNT (Tris, sodium, Tween 20) at 37 °C for 40 min. The chips were stained by streptavidin–alexa647 (1:500) dilution in TNT for 30 min. Post-staining washing was conducted in 1× TNT FOR 40 min.

## 4. Conclusions

Taking account that RSV enhances residual *CPT2* activities in human fibroblasts derived from *CPT2*-deficient patients and restores normal fatty acid oxidation rates, we now report changes in miRNA expression linked to *CPT2*-deficiency, and we identify miRNAs whose expression changed following RSV treatment of control or *CPT2*-deficient fibroblasts isolated from patients. Our findings suggest that RSV consumption might exert beneficiary effects in patients with *CPT2*-deficiency through miRNAs expression modulation.

## Figures and Tables

**Table 1:** MiRNAs whose expression changed in human *CPT2*-deficient primary fibroblasts as compared with control primary human fibroblasts, as deduced from microRNA microarray analysis. Geometric mean of intensities <100 were considered as background and discarded. Changes were considered significant for *p* < 0.05.

miRNAs	Fold Change	Increasing Parametric *p* Value
miRNAs upregulated in *CPT2*-deficient fibroblasts:		
483	3.1	1.6 × 10^−6^
301	11.43	4.1 × 10^−6^
449b	1.99	2.79 × 10^−6^
206	3.38	9.39 × 10^−6^
550-1	2.83	0.000171
539	2.04	0.0002213
661	2.79	0.0004408
371	2.65	0.0005968
10b	2.75	0.0011091
9	4.4	0.0014253
550-2	2.1	0.0016581
651	2.87	0.0019172
196a-2	2.09	0.0019172
miRNAs downregulated in *CPT2*-deficient fibroblasts:		
let-7d	0.16	<1 × 10^−7^
211	0.14	4 × −10^−7^
let-7a3	0.22	1.2 × 10^−6^
198	0.14	2.8 × 10^−6^
141	0.28	4.6 × 10^−6^
136	0.31	5.1 × 10^−6^
203	0.24	5.7 × 10^−6^
127	0.23	7.7 × 10^−6^
181c	0.26	1.85 × 10^−5^
496	0.3	2.5 × 10^−5^
126-5p	0.14	3.64 × 10^−5^
144	0.097	3.8 × 10^−5^
let-7g	0.48	4.14 × 10^−5^
181a2	0.44	4.42 × 10^−5^
618	0.48	4.47 × 10^−5^
41	0.15	4.5 × 10^−5^
299-5p	0.14	4.73 × 10^−5^
1	0.41	4.88 × −10^−5^
145	0.32	4.94 × 10^−5^
25	0.26	6.37 × 10^−5^
123	0.31	6.67 × 10^−5^
200b	0.27	8.33 × 10^−5^
325	0.44	8.51 × 10^−5^
593	0.42	9.19 × 10^−5^
24-5p/189	0.14	0.0001524
125b2	0.1	0.0002071
123	0.25	0.0002196
154-5p	0.3	0.0002281
184	0.43	0.0002499
199b	0.47	0.0005099
22	0.25	0.0006033
363-3p	0.37	0.0006076
338	0.24	0.0007282
146a	0.42	0.0008154
212	0.28	0.0008813
196a-1	0.34	0.0008916
500	0.29	0.0013401
563	0.47	0.0016458

**Table 2:** RSV (75 μM) treatment impacts miRNA expression in control and in *CPT2*-deficient fibroblasts, compared with the corresponding DMSO-treated primary fibroblasts, as deduced from microRNA microarray analysis. Geometric mean of intensities <100 were considered as background and discarded. Changes were considered significant when *p* < 0.05.

Control Fibroblasts						*CPT2*-Deficient Fibroblasts					
Upregulation by RSV			Downregulation by RSV			Upregulation by RSV			Downregulation by RSV		
miRNA	Fold Change	Increasing Parametric *p* Value	miRNA	Fold Change	Increasing Parametric *p* Value	miRNA	Fold Change	Increasing Parametric *p* Value	miRNA	Fold Change	Increasing Parametric *p* Value
321	3.67	0.0003277	35	0.47	0.0011099	219	1.81	0.00028111	101-1/2	0.51	0.000758
594	3.33	0.000695	548a-1	0.28	0.0011964	299-5p	1.94	0.00037058	181d	0.45	0.00021178
550-2	2.65	0.0026216	566	0.49	0.0014556	193a	1.96	0.00074255	16-1	0.48	0.00022522
565	2.87	0.0066109	620	0.49	0.0031365	199a1-5p	1.8	0.035782	21	0.47	0.00023066
611	2.18	0.0100121	92b	0.24	0.003498	548a1	2.3	0.041391	99a*	0.27	0.00041291
483	2.29	0.0118661	378-5p	0.41	0.0070053	337	1.89	0.0488272	20b	0.47	0.00053956
335	2.69	0.0158687	579	0.19	0.020456				let-7d	0.46	0.00071834
550-1	2.35	0.0182839	136	0.46	0.0206073				17-5p	0.43	0.0007255
449b-1	1.99	0.021485	let-7f	0.44	0.0220297				146a	0.27	0.0110431
661	2.45	0.0256413	211	0.24	0.0220881				566	0.5	0.012436
326	3.58	0.0315627	376a-2	0.42	0.024334				376b	0.47	0.012361
196a-1	3.45	0.0369082	193a	0.23	0.0255173				26a	0.46	0.0130816
			29a	0.47	0.0265262				103-1	0.35	0.0166397
			199b	0.44	0.0294717				let-7c	0.28	0.0192349
			141	0.26	0.029536				423	0.14	0.0231317
			204	0.33	0.0295895				23a	0.18	0.0257826
			216	0.3	0.0311748						
			let-7a3	0.29	0.0323893						
			618	0.44	0.0361713						
			198	0.43	0.0375283						
			22	0.39	0.0379847						
			126-5p	0.26	0.0463448						
			23b	0.49	0.0469568						
			144	0.26	0.0496528						

**Table 3:** Putative targets transcripts of miRNAs of Table 1 and Table 2.

Genes	Proteins	MiRNAs *
**Putative target transcripts of miRNAs upregulated in *CPT2*-deficient fibroblasts:**		
*SIRT1*	SIRT1	2 × 449b/539/9/651/
*STK11*	LKB1	483/
*PRKAA1*	AMPK subunit	301/449b/539/371/9/651/
*PRKAA2*	AMPK subunit	483/301/2 × 449b/206/4 × 371/3 × 10b/3 × 651/
*PRKAB1*	AMPK subunit	483/301/9/
*PPARGC1A*	PGC-1 α	2 × 301/539/196a-2/
*ALDH7A1*	PDE	2 × 483/449b/2 × 371 2 × 10b/651/
*ESR1*	ER	483/3 × 301/2 × 206/ 371/2 × 9/196a-2/
*ESRRA*	ERR α	449b/
*NRF1*	NRF1	483/449b/2 × 539/3 × 371/9/
*NFE2L2*	NRF2	651/
*TFAM*	TFAM	483/206/539/2 × 371/10b/9/651/
**Putative target transcripts of miRNAs downregulated in *CPT2*-deficient fibroblasts:**		
*SIRT1*	SIRT1	211/141/136/181a2,c/496/126-5p/
*STK11*	LKB1	-
*PRKAA1*	AMPK subunit	496/126-5p/144/
*PRKAA2*	AMPK subunit	let-7a3,d,g/2 × 141/203/3 × 181a2,c/4 × 126-5p/144/9/
*PRKAB1*	AMPK subunit	2 × 141/2 × 203/181a2,c/
*PPARGC1A*	PGC-1 α	let-7a3,d,g/211/141/136/203/496/3 × 126-5p/144/
*ALDH7A1*	PDE	141/2 × 136/203/
*ESR1*	ER	211/136/203/181a2,c/496/
*ESRRA*	ERR α	-
*NRF1*	NRF1	2 × 211/181a2,c/
*NFE2L2*	NRF2	181a2,c/496/
*TFAM*	TFAM	211/4 × 141/2 × 136/2 × 203/181a2,c/496/4 × 126-5p/4 × 144/
**Putative target transcripts of miRNAs upregulated after RSV treatment of *CPT2*-deficient fibroblasts:**		
*SIRT1*	SIRT1	199a1-5p/
*STK11*	LKB1	199a1-5p/
*PRKAA1*	AMPK subunit	-
*PRKAA2*	AMPK subunit	219/2 × 299/193a/199a1-5p/2 × 337/
*PRKAB1*	AMPK subunit	193a/
*PPARGC1A*	PGC-1 α	219/193a/2 × 199a1-5p/
*ALDH7A1*	PDE	199a1-5p/2 × 337/
*ESR1*	ER	219/299/2 × 193a/337/
*ESRRA*	ERR α	-
*NRF1*	NRF1	199a1-5p/
*NFE2L2*	NRF2	337/
*TFAM*	TFAM	299-5p/2 × 193a/199a1-5p/2 × 337/
**Putative target transcripts of miRNAs downregulated after RSV treatment of *CPT2*-deficient fibroblasts:**		
*SIRT1*	SIRT1	181d/23a/
*STK11*	LKB1	20b/17-5p/
*PRKAA1*	AMPK subunit	2 × 101-1/2/16-1/21/26a/
*PRKAA2*	AMPK subunit	3 × 181d/21/20b/let-7c,d/17-5/2 × 146a/376b/26a/23a/
*PRKAB1*	AMPK subunit	181d/146a/
*PPARGC1A*	PGC-1α	101-1/2/let-7d/376b/26a/2 × 23a/
*ALDH7A1*	PDE	16-1/2 × 20b/2 × 17-5p/146a/
*ESR1*	ER	181d/21/3 × 20b/3 × 17-5p/146a/2 × 26a/2 × 103-1/23a/
*ESRRA*	ERRα	16-1/103-1/423/
*NRF1*	NRF1	181d/2 × 21/
*NFE2L2*	NRF2	181d/103-1/
*TFAM*	TFAM	181d/3 × 20b/3 × 17-5p/2 × 376b/26a/23a/

* MiRNAs are given in the same order as in Table 1 and Table 2. The sign “x“ indicates the number of putative target sequence for a given miRNA. Target transcripts were identified using the Targetscan software (www.targetscan.com). Numbers in front of miRNAs indicate that more than one consensus target site for this miRNA is present in the 3′-untranslated region of the transcript. For instance, *SIRT1* 3-untranslated region contains two consensus target sequences for *miR-449b*.
